# ALSGate: An Efficient Gated Mixture‐of‐Experts Model for Reliable ALS Detection Using EMG Signals

**DOI:** 10.1049/htl2.70097

**Published:** 2026-09-03

**Authors:** Sandipa Chowdhury, Sudipto Pramanik, Sudha Bhattacharjee, Motasim Billah

**Affiliations:** ^1^ Department of Electrical and Electronic Engineering Bangladesh University of Engineering and Technology Dhaka Bangladesh; ^2^ Department of Electrical and Electronic Engineering Daffodil International University Dhaka Bangladesh; ^3^ Department of Computer Science and Engineering Daffodil International University Dhaka Bangladesh

**Keywords:** amyotrophic lateral sclerosis (ALS), electromyography (EMG), exponential moving average (EMA), focal loss, mixture of experts (MoE), spectrogram‐based CNN, temporal convolutional network (TCN)

## Abstract

Amyotrophic lateral sclerosis (ALS) is a progressive neurodegenerative disorder affecting motor neurons, resulting in neuromuscular weakness and paralysis. Electromyography (EMG) is of vital importance for the detection of ALS. In this paper, a refined mixture of experts is proposed that automatically discriminates ALS patients from non‐ALS cases using clinical EMG signals from the N2001 EMGLAB open‐access dataset. The architecture consists of a 1D convolutional neural network, a temporal convolutional network and a spectrogram‐based CNN to collectively learn localised temporal, long‐range temporal and spectral features from EMG activity. A gating mechanism dynamically weights expert contributions and performs significantly better than equal‐weight fusion. Training with focal loss and exponential moving average stabilisation addresses class imbalance and improves convergence. The proposed approach reached an AUROC of 0.9992, an F1‐score of 0.9903 and a balanced accuracy of 0.9905, demonstrating strong discriminative performance and potential for real‐time clinical applications.

## 1 Introduction

Amyotrophic Lateral Sclerosis (ALS) is a rapidly progressing neurodegenerative disorder leading to muscle weakness, paralysis and eventual respiratory failure [1]. Due to overlapping symptoms with other neuromuscular disorders, timely and accurate diagnosis remains challenging yet essential [2]. Electromyography (EMG) is a key diagnostic tool for detecting denervation, reinnervation and motor unit changes, often before clinical symptoms manifest, making it central to ALS screening and decision support research [3].

Initial studies demonstrated that EMG contains discriminative features in the time, frequency and time–frequency domains [4, 5]. For instance, tunable‐Q wavelet transforms have been used to extract robust spectral‐statistical features that distinguish ALS from healthy or myopathic signals [6]. As data volume and computational power have grown, approaches have shifted from handcrafted feature engineering to deep learning–based feature discovery [7]. Models such as spectrogram‐based 2D CNNs and raw‐signal 1D CNNs now learn discriminative patterns directly from EMG, often outperforming manual pipelines [8, 9, 10]. Boutellaa et al. compared CNNs, LSTMs and ConvLSTM hybrids on EMGLAB data and reported promising results [11].

Despite this progress, important limitations remain. Most recent studies rely on a single deep learning architecture without adaptive hybrid modelling across temporal and spectral representations, which may limit generalisation across subjects, muscles and noisy signal conditions. For example, ResEMGNet used a static CNN on raw EMG for ALS and myopathy detection, with no adaptability to signal variability [12], while VGG16 applied to EMG spectrograms lacked dynamic feature fusion [13]. To the best of our knowledge, recent EMG‐based ALS detection studies have not explored mixture of experts (MoE) or adaptive expert fusion, despite their success in other biomedical AI applications [14]. Likewise, training strategies such as mixup, time masking, exponential moving average (EMA) and test‐time augmentation (TTA) remain underutilised in EMG ALS detection, even though they are increasingly common in other biomedical time‐series tasks [15]. Furthermore, although loss functions are central to model optimisation, ALS EMG research largely depends on standard cross‐entropy, with limited exploration of adaptive alternatives such as focal loss.

**FIGURE 1:**
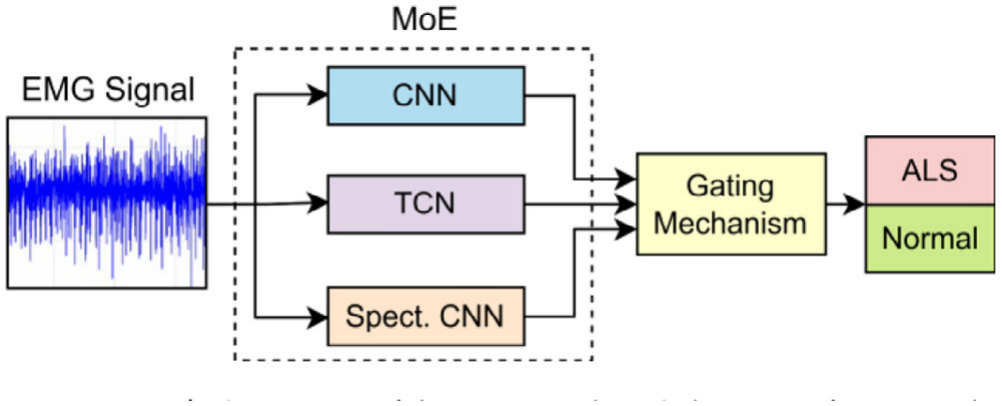
Overview of the proposed ALS detection framework.

To address these gaps, this work proposes the framework shown in Figure 1. The model introduces a novel MoE architecture for ALS detection by combining three expert branches: a raw‐signal CNN, a temporal TCN and a spectrogram‐based CNN. This design enables adaptive fusion of local temporal, long‐range temporal and frequency‐domain information. The study also systematically evaluates different loss functions, including standard BCE, weighted BCE and focal loss, to improve learning under class imbalance and hard‐sample variability. In addition to deep features, the model incorporates normalised statistical EMG biomarkers such as zero‐crossing rate (ZCR), root mean square (RMS) and waveform length (WL) to enhance generalisation while preserving physiological interpretability. Modern training strategies such as EMA and TTA are also incorporated. Finally, unlike prior work that may risk data leakage through window‐level evaluation, this study adopts subject‐level stratified splitting to ensure more realistic and unbiased performance assessment.

## 2 Methodology

**FIGURE 2:**
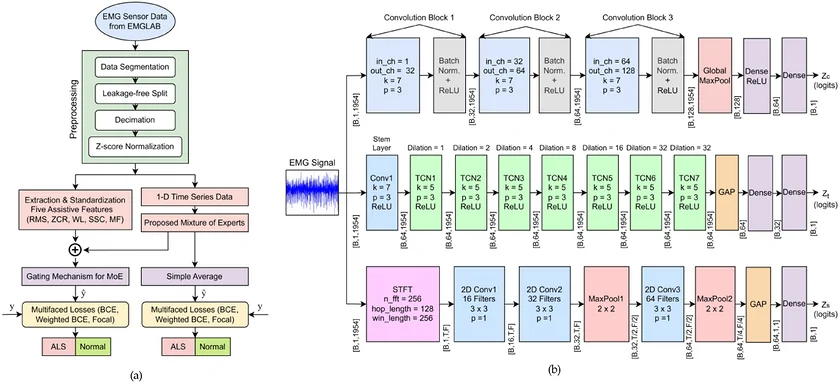
(a) Proposed framework for ALS detection; (b) mixture of experts architecture.

This section presents the proposed framework for automatically distinguishing ALS subjects from healthy subjects using clinical EMG signals. The overall workflow is shown in Figure 2a. It includes three specialised experts, a gating mechanism for flexible fusion and multiple loss functions for robust optimisation.

### 2.1 Proposed Mixture of Experts Model

Three complementary experts are integrated in the proposed model, each intended to capture a distinct characteristic of the EMG signal: (i) a 1D CNN for local temporal analysis, (ii) a temporal convolutional network (TCN) for long‐range temporal dependency modelling and (iii) a spectrogram‐based CNN for time–frequency representation. Figure 2b shows the detailed architecture of these three experts.

#### 2.1.1 CNN Expert

The CNN expert employs three stacked one‐dimensional convolutional layers to learn localised temporal representations from raw EMG signals. This branch is designed to capture short‐duration neuromuscular signatures, such as positive sharp waves, fasciculation potentials and fibrillation potentials. Each convolutional layer is followed by batch normalisation and ReLU activation. The resulting feature maps are compressed using adaptive global max pooling, which retains the strongest temporal activations. The pooled representation is projected through a fully connected layer with nonlinear activation and subsequently mapped to a single output logit $Z_{c}$ for expert fusion.

#### 2.1.2 TCN Expert

The TCN expert is designed to capture long‐range temporal dependencies in EMG signals. This is important because motor unit remodelling in ALS results in prolonged and polyphasic motor unit potentials (MUPs) that evolve over extended temporal intervals. An initial convolutional stem first projects the raw EMG signal into a 64‐dimensional feature space, followed by seven residual TCN blocks with progressively increasing dilation factors. Each residual block contains two dilated convolutional layers with kernel size 5, followed by batch normalisation and nonlinear activation, with dropout applied for regularisation. The dilated convolutions enlarge the receptive field, while residual connections facilitate stable gradient propagation. Finally, adaptive global average pooling aggregates the temporal feature representations into a fixed‐length vector, which is passed through a fully connected layer to generate the TCN logit $Z_{t}$.

#### 2.1.3 Spectrogram CNN Expert

To capture frequency‐dependent characteristics of EMG activity, the spectrogram CNN expert converts the one‐dimensional EMG signal into a logarithmic magnitude spectrogram using the Short‐Time Fourier Transform. A Hann window with an FFT size of 256, window length of 256 and hop length of 128 is used for time–frequency representation. This transformation enables the model to exploit spectral variations associated with ALS‐related motor unit alterations, where chronic reinnervation may produce shifts in frequency distribution due to prolonged MUP durations. The generated spectrogram is processed by a 2D CNN consisting of three convolutional layers with 16, 32 and 64 filters, respectively. Each convolutional layer is followed by batch normalisation and ReLU activation, while max‐pooling layers progressively reduce the spatial dimensions. Finally, adaptive global average pooling compresses the feature maps into a fixed‐length representation, which is passed through a linear layer to generate the spectrogram expert logit $Z_{s}$.

Each expert captures a unique and complementary aspect of ALS‐related EMG pathology. Their combination enables a more comprehensive interpretation of the signal.

### 2.2 Gating Mechanism

**FIGURE 3:**
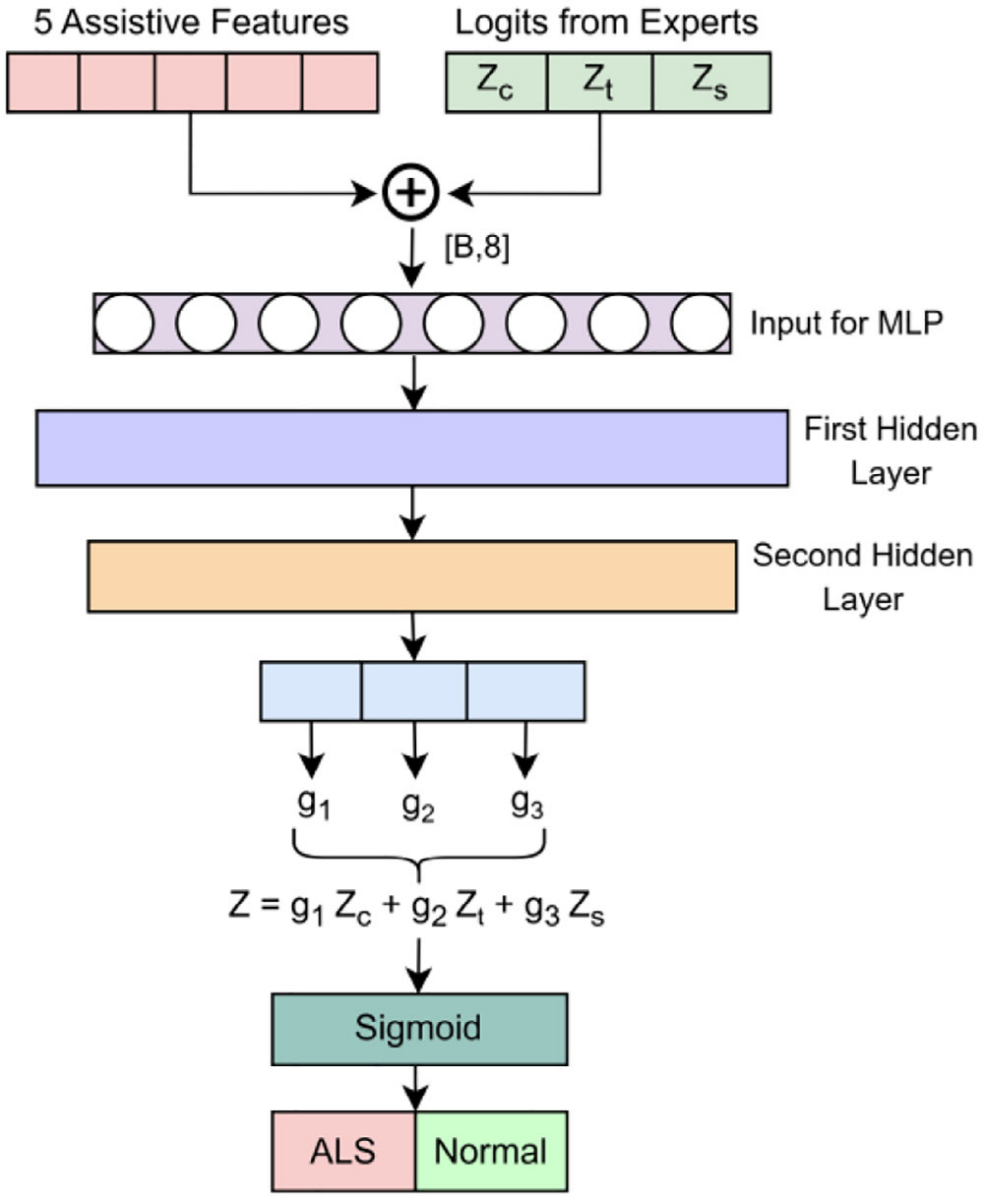
Gating mechanism.

Expert fusion is controlled by a gating mechanism that learns data‐driven weights rather than relying on static averaging. Five handcrafted assistive features are extracted from each EMG window and concatenated with the three expert logits ($Z_{c}$, $Z_{t}$ and $Z_{s}$) to improve fusion decisions. Figure 3 illustrates the gating mechanism.

#### 2.2.1 Handcrafted Features

Auxiliary physiological context is provided by the handcrafted features, which aid in the gating mechanism's interpretation of signal variability. Five features are extracted from each EMG segment:

- **RMS**: Quantifies overall muscle activity; Lower RMS values indicate muscle weakness, typical in ALS.
- **ZCR**: Counts polarity changes in the signal; High ZCR suggests frequent twitches or spasms.
- **Waveform length (WL)**: Measures signal roughness; Elevated WL reflects non‐smooth or erratic muscle movements.
- **Slope sign change (SSC)**: Detects rapid direction changes in slope, indicative of motor instability.
- **Mean frequency (MF)**: Represents the spectral centroid; A reduction in MF indicates degenerative muscle behaviour.

These descriptors $\left(x_{\mathrm{tab}}\right)$ supplement the deep features learned by the experts and guide the gating network toward more physiologically meaningful weighting.

#### 2.2.2 Dynamic Weight Assignment

A two‐layer perceptron (MLP) receives the concatenated vector [𝑍_𝑐_, 𝑍_𝑡_, 𝑍_𝑠_, 𝑥_𝑡𝑎𝑏_] and produces three scalar weights (𝑔_1_, 𝑔_2_, 𝑔_3_). A softmax activation ensures that the normalised gating weights sum to one:

(1) $$\;g_{i}=\frac{e^{h_{i}}}{\sum_{j=1}^{3}e^{h_{j}}}\;,\;\;\sum_{i=1}^{3}g_{i}=1$$

where *h_i_* are the pre‐activation outputs of the MLP. The final fused logit is computed as:

(2) $$Z=g_{1}Z_{c}+g_{2}Z_{t}+g_{3}Z_{s}$$

This adaptive weighting enables the model to emphasise the most relevant expert depending on the input characteristics.

#### 2.2.3 Comparison With Equal‐Weight MoE

For comparison, an equal‐weight MoE baseline is defined as:

(3) $$Z=\frac{1}{3}\left(Z_{c}+Z_{t}+Z_{s}\right)$$

where each expert contributes equally to the final prediction. Although simpler, this method lacks flexibility under varying input dynamics. In contrast, the proposed gated MoE adapts to the signal characteristics and can improve generalisation across heterogeneous EMG samples.

### 2.3 Multifaceted Loss Functions

Binary cross‐entropy (BCE), weighted BCE and Focal Loss are used to optimise ALS versus normal discrimination under class imbalance and variable sample difficulty.

#### 2.3.1 Binary Cross‐Entropy (BCE)

(4) $$L_{BCE}=\frac{1}{N}\sum_{i}^{N}\left[y_{i}log\left({\hat{y}}_{i}\right)+\left(1-y_{i}\right)log\left(1-{\hat{y}}_{i}\right)\right]$$

This is the baseline objective for probabilistic binary classification

#### 2.3.2 Weighted BCE

(5) $$\;L_{\mathrm{WBCE}}=-\frac{1}{N}\sum_{i=1}^{N}w_{i}\;\left[y_{i}\log\left({\hat{y}}_{i}\right)+\left(1-y_{i}\right)\log\left(1-{\hat{y}}_{i}\right)\right]$$

This variant uses class or sample weights $w_{i}$ to counter class imbalance.

#### 2.3.3 Focal Loss

(6) $$\;L_{\mathrm{Focal}}=-\frac{1}{N}\sum_{i=1}^{N}\alpha \;\left[y_{i}{\left(1-{\hat{y}}_{i}\right)}^{\gamma }\log\left({\hat{y}}_{i}\right)+\left(1-y_{i}\right){{\hat{y}}_{i}}^{\gamma }\log\left(1-{\hat{y}}_{i}\right)\right]$$

Focal Loss down‐weights easy samples and focuses learning on hard or ambiguous cases through the focusing parameter γ, while α balances class importance

## 3 Experimental Details

### 3.1 Dataset

The N2001 EMGLAB clinical EMG dataset [16] was used, restricting the analysis to the normal and ALS cohorts (10 normal subjects and 8 ALS subjects). Needle‐EMG was recorded from the biceps brachii and vastus medialis using a concentric needle electrode under standard motor unit action potential acquisition conditions. Signals were sampled at 24 kHz for approximately 11s. A total of 302 signals were used (151 normal and 151 ALS).

### 3.2 Data Preprocessing

To ensure consistency, balance and feature readiness for deep learning, the EMG signals underwent several preprocessing steps. Each signal was segmented into eleven non‐overlapping 1 s windows, yielding 302×11 = 3322 samples. Subject‐disjoint training, validation and test splits were used in a 70%, 15% and 15% ratio, respectively. Z‐score normalisation was applied to each EMG window. Welch's power spectral density (PSD) estimate was used to obtain the frequency‐domain feature, while the remaining hand‐crafted features were extracted directly from the time‐domain EMG signals. The extracted features were then standardised using *StandardScaler* before being provided to the gating network.

### 3.3 Experimental Framework

The experimental setup was designed to compare multiple deep learning models for EMG‐based ALS classification under controlled and reproducible conditions. All experiments were conducted in PyTorch with fixed random seeds. Two model variations were tested: (1) the proposed MoE model with adaptive gating and (2) an equal‐weight MoE baseline. Each model processed normalised 1D EMG windows with optional handcrafted features and produced a binary prediction for ALS or non‐ALS.

Training used three loss functions: BCE, weighted BCE based on inverse class frequency and Focal Loss with α = 0.25 and γ = 2.0. The models were optimised using AdamW with learning rate of 3$\;\times 10^{-4}$ and weight decay of 1$\;\times 10^{-4}$ for up to 20 epochs. Early stopping was based on validation AUROC with a patience of 3 and a minimum change threshold of 5$\;\times 10^{-4}$. Stabilisation techniques included gradient clipping with a maximum norm of 5.0, EMA with a decay of 0.999 and mixed‐precision training. During inference, TTA = 5, using noise and jitter was applied. Performance was measured using AUROC, AUPRC, F1‐score and balanced accuracy. The optimal classification threshold was selected on the validation set by maximising Youden's index and the selected threshold was subsequently applied to the test set for final evaluation.

## 4 Results and Discussion

To assess the effectiveness of the proposed Mixture‐of‐Experts framework, the performance of the *gated MoE* and the *equal‐weight MoE* models was evaluated using the three previously defined loss functions (Table 1). All configurations achieved strong results, confirming the capability of the proposed framework to capture ALS‐specific EMG patterns.

**TABLE 1:** Comparative evaluation between gated mixture‐of‐experts and equal weight mixture‐of‐experts models.

	BCE				Weighted BCE				Focal			
Model	Precision	Recall	F1	ACC	Precision	Recall	F1	ACC	Precision	Recall	F1	ACC
**Gated MoE**	**0.9933**	**0.9613**	**0.9770**	**0.9778**	**1.000**	**0.9774**	**0.9819**	**0.9823**	**0.9967**	**0.9839**	**0.9903**	**0.9905**
Eq. W. MoE	0.9769	0.9548	0.9657	0.9676	0.9774	0.9645	0.9774	0.9789	0.9967	0.9710	0.9837	0.9841

Performance consistently improved from BCE to weighted BCE and then to focal loss. Standard BCE treats all samples equally and may bias the model toward the majority class. Weighted BCE compensates for class imbalance by assigning higher penalties to minority‐class samples and thus improves recall. Focal loss provides the best trade‐off by emphasising hard examples and improving discrimination, yielding an AUROC of 0.9992, F1‐score of 0.9903 and a balanced accuracy of 0.9905 for the gated MoE model.

The *gated MoE* outperformed the *equal‐weight MoE* across all loss settings, with the most notable gain under weighted BCE. The adaptive gating mechanism dynamically regulates expert contributions and suppresses antagonistic or low‐confidence predictions, thereby reducing false positives and improving clinical reliability.

To examine the contribution of statistical features, five hand‐crafted assistive features (RMS, ZCR, WL, SSC, MF) were incorporated into the gating network. As shown in Table 2, the inclusion of these features improved both F1‐score and accuracy under BCE loss for the gated MoE. These descriptors provided complementary global context to the expert logits and improved robustness.

**TABLE 2:** Effect of assistive features (BCE loss, gated MoE).

Configuration	AUROC	AUPRC	F1	ACC
**With assistive features**	**0.9933**	**0.9613**	**0.9770**	**0.9778**
Without assistive features	0.9867	0.9581	0.9722	0.9734

The proposed gated MoE framework also outperformed several existing approaches reported in the literature on the N2001.

EMGLAB dataset, as shown in Table 3. These results indicate that adaptive fusion of complementary temporal, spectral and handcrafted features can improve ALS discrimination from EMG recordings.

**TABLE 3:** Performance comparison of the proposed framework with existing methods from the literature.

Authors	Methodology	Dataset	ACC (%)
Mokdad et al. [4]	CWT, SVM	EMGLAB [16]	95.80
Hassan et al. [9]	ALSNet	EMGLAB [16]	97.74
Parusheva et al. [10]	CNN‐Attention	EMGLAB [16]	98.49
Makam et al. [5]	SSA, PSO, QCNN	EMGLAB [16]	98.50
**Proposed method**	Gated MoE	EMGLAB [16]	**99.05**

## 5 Conclusion

Clinical EMG signals provide a strong basis for the early detection of neuromuscular diseases such as ALS because they offer direct electrophysiological insight into motor unit activity. This work introduced an enhanced MoE frame‐work that combines local temporal, long‐range temporal and spectral representations through an adaptive gating mechanism for reliable ALS detection. The gating network learns expert relevance dynamically across heterogeneous EMG patterns, while handcrafted assistive features enhance interpretability and generalisation. The proposed model achieves high classification performance while maintaining computational efficiency. Overall, the framework improves the reliability of EMG‐based ALS detection and shows strong promise for clinical decision support systems.

## Author Contributions

**Sandipa Chowdhury**: conceptualisation, data curation, formal analysis, investigation, methodology, software, validation, writing – original draft. **Sudipto Pramanik**: conceptualisation, formal analysis, investigation, methodology, validation, visualisation, writing – original draft, writing – review and editing. **Sudha Bhattacharjee**: conceptualisation, investigation, methodology, validation, writing – review and editing. **Motasim Billah**: data curation, investigation, resources, writing – review and editing.

## Funding

The authors have nothing to report.

## Conflicts of Interest

The authors declare no conflicts of interest.

## Data Availability

The data that support the findings of this study are openly available in the EMGLAB repository at http://www.emglab.net. The Google Colab notebook containing the complete implementation for data preprocessing, model training and performance evaluation is publicly available at https://github.com/sandipaeee/ALSGate‐EMG‐Classification.

## Appendix A Mathematical Definitions of the Assistive EMG Features

### A.1

The proposed gating network incorporates five handcrafted EMG features to provide complementary physiological information alongside the expert logits. Let an EMG window be represented as

(A1)
$$X=\left\{x\left[1\right],x\left[2\right],\text{…},x\left[N\right]\right\},$$

where N denotes the number of samples in the EMG segment. The following time‐domain and frequency‐domain features are extracted from each normalised EMG window.

### A.1 Root Mean Square

The RMS quantifies the signal amplitude and overall energy level of the EMG activity. It is calculated as

(A2)
$$\mathrm{RMS}=\sqrt{\frac{1}{N}\sum_{n=1}^{N}x^{2}\left[n\right]}$$

A higher RMS value indicates increased signal amplitude, whereas lower RMS values may reflect reduced muscle activation

### A.2 Zero‐Crossing Rate

The ZCR measures the number of polarity changes in the EMG waveform. It is computed as

(A3)
$$\mathrm{ZCR}\;=\frac{1}{N-1}\sum_{n=1}^{N-1}I\;\left(x\left[n\right]x\left[n+1\right]<0\right)$$

where I(·) denotes the indicator function. This feature captures variations related to the oscillatory behaviour and frequency characteristics of the EMG signal.

### A.3 Waveform Length

Waveform length (WL) represents the average cumulative variation of the EMG waveform and is calculated as

(A4)
$$\mathrm{WL}\;=\frac{1}{N-1}\sum_{n=2}^{N}\left|x\left[n\right]-x\left[n-1\right]\right|$$

Higher WL values indicate increased waveform complexity and rapid signal fluctuations.

### A.4 Slope Sign Change

SSC measures changes in the direction of the signal slope. In this study, SSC is calculated without an amplitude threshold and is defined as

(A5)
$$\mathrm{SSC}\;=\frac{1}{N-2}\sum_{n=2}^{N-1}I\;\left(\left(x\left[n+1\right]-x\left[n\right]\right)\left(x\left[n\right]-x\left[n\;-1\right]\right)<0\right)$$

This feature reflects rapid changes in the temporal morphology of the EMG waveform.

### A.5 Mean Frequency

The frequency‐domain feature is obtained using Welch's PSD estimation. Let 𝑃[𝑓*_k_*] denote the estimated PSD at frequency 𝑓_𝑘_. The mean frequency (MF) represents the spectral centroid of the EMG power spectrum and is calculated as

(A6)
$$\mathrm{MF}\;=\frac{\sum_{k=1}^{K}f_{k}P\left[f_{k}\right]}{\sum_{k=1}^{K}P\left[f_{k}\right]}$$

The MF provides information about the distribution of spectral energy within the EMG signal and can reflect frequency shifts associated with changes in motor unit activity.

The extracted handcrafted features are combined into a feature vector:

(A7)
$$x_{\mathrm{tab}}={\left[\text{RMS},\text{ZCR},\text{WL},\text{SSC},\text{MF}\right]}^{T}$$

Before being supplied to the gating network, each feature is standardised using statistics calculated only from the training partition. For feature j of sample i, the standardised value is

(A8)
$$\;{\tilde{x}}_{i,j}=\frac{x_{i,j}-\mu_{j}^{\mathrm{train}}}{\sigma_{j}^{\mathrm{train}}+\varepsilon }$$

where 𝜇_𝑗_𝑡𝑟𝑎𝑖𝑛 and 𝜎_𝑗_𝑡𝑟𝑎𝑖𝑛 represent the mean and standard deviation obtained from the training data and 𝜖 is a small constant for numerical stability. The same training statistics are applied to validation and test samples.

## Appendix B Training and Inference Procedure

### B.1 Window‐Level Signal Normalisation

Each one‐second EMG window is independently normalised using z‐score normalisation. For an EMG window x, the normalised signal is calculated as

(B1)
$$x_{\mathrm{norm}}\;\left[n\right]=\frac{x\left[n\right]-\mu_{x}}{\sigma_{x}+\varepsilon }\;$$

where $\mu_{x}$ and $\sigma_{x}$ represent the sample mean and standard deviation of the corresponding EMG window, respectively.

### B.2 Forward Computation

For each normalised EMG window, the CNN, TCN and spectrogram‐based CNN experts generate logits $Z_{c}$, $Z_{t}$ and $Z_{s}$, respectively. These logits are concatenated with the standardised assistive feature vector:

(B2)
$$v={\left[{\tilde{x}}_{\mathrm{tab}},Z_{c},Z_{t},Z_{s}\right]}^{T}\;$$

The gating multilayer perceptron maps this eight‐dimensional vector to three unnormalised gating scores:

(B3)
$$h=G_{\phi }\;\left(v\right)$$

where $G_{\phi }\left(\cdot \right)$ denotes the gating network.

The normalised expert weights are obtained using softmax:

(B4)
$$\;g_{m}=\frac{\exp\left(h_{m}\right)}{\sum_{r=1}^{3}\exp\left(h_{r}\right)}\;,\;m\;\in \;\left\{1,\;2,\;3\right\}$$

The fused output logit is computed as

(B5)
$$Z=g_{1}Z_{c}+g_{2}Z_{t}+g_{3}Z_{s}$$

The predicted probability of ALS is obtained using the sigmoid activation:

(B6)
p=σ(Z)=1+exp(−Z)

### B.3 Model‐Training Procedure

The training procedure of the proposed gated MoE model is summarised as follows:

1. Each EMG recording is segmented into eleven non‐overlapping one‐second windows.
2. Each EMG window is normalised using window‐level z‐score normalisation.
3. RMS, ZCR, WL, SSC and MF features are extracted from each EMG window.
4. The assistive features are standardised using statistics estimated from the training partition and the same transformation is applied to validation and test partitions.
5. Each normalised EMG window is supplied to the CNN, TCN and spectrogram‐based CNN to obtain $Z_{c}$, $Z_{t}$ and $Z_{s}$.
6. The three expert logits are concatenated with the five standardised assistive features and provided to the gating network.
7. The fused logit and ALS probability are calculated using the adaptive gating mechanism.
8. The model is optimised using BCE, weighted BCE, or focal loss.
9. Mixed‐precision training and gradient clipping with a maximum norm of 5.0 are applied during backpropagation. The model parameters are updated using AdamW optimisation.
10. An EMA model is maintained during training.
11. The EMA model is evaluated on the validation partition after each epoch.
12. Training terminates when validation AUROC does not improve by at least $5\times 10^{-4}$ for three consecutive epochs, or when the maximum number of 20 epochs is reached.

### B.4 Exponential Moving Average

To improve training stability, an EMA of the model parameters is maintained. Let 𝜃_𝑡_ denote the model parameters after optimisation step t and let $\theta_{t}^{\mathrm{EMA}}$ denote the corresponding EMA parameters. The update is defined as

(B7)
$$\;\theta_{t}^{\mathrm{EMA}}=\beta \theta_{t-1}^{\mathrm{EMA}}+\left(1-\beta \right)\theta_{t}$$

where *β* = 0.999 is the EMA decay coefficient.

Validation and test predictions are generated using the EMA model.

### B.5 Test‐Time Augmentation

During inference, five augmented versions of each EMG window are evaluated. The transformations include additive noise and temporal jitter. The final prediction probability is obtained by averaging the probabilities from all augmented samples:

(B8)
$$p=\frac{1}{K_{\mathrm{TTA}}}\;\sum_{k=1}^{K_{\mathrm{TTA}}}\sigma \left(F_{\theta }\left(T_{k}\left(x\right)\right)\right)$$

where 𝐾_TTA_ = 5 and $F_{\theta }$ represents the EMA model.

### B.6 Validation‐Based Decision Threshold

The binary classification threshold is selected using only the validation partition. The optimal threshold is obtained by maximising Youden's index:

(B9)
$$\;\tau^{*}=\arg\max_{\tau }\left[\mathrm{Sensitivity}\left(\tau \right)+\mathrm{Specificity}\left(\tau \right)-1\right]\;$$

The selected threshold is then fixed and applied to the test partition:

(B10)
$$\;\hat{y}=\left\{\begin{array}{c} 1,\;p\geq \tau^{*}\\ 0,\;p<\tau^{*} \end{array}\right.\;$$
